# Prevalence of smoking and incidence of initiation in the Latin American adult population: the PLATINO study

**DOI:** 10.1186/1471-2458-9-151

**Published:** 2009-05-22

**Authors:** Ana M Menezes, Maria V Lopez, Pedro C Hallal, Adriana Muiño, Rogelio Perez-Padilla, José R Jardim, Gonzalo Valdivia, Julio Pertuzé, Maria M de Oca, Carlos Tálamo, Cesar G Victora, the PLATINO Team

**Affiliations:** 1 Federal University of Pelotas, Pelotas, Brazil; 2 Universidad de la República, Montevideo, Uruguay; 3 Instituto Nacional de Enfermedades Respiratorias, Mexico City, Mexico; 4 Federal University of São Paulo, São Paulo, Brazil; 5 Pontifícia Universidad Católica de Chile, Santiago, Chile; 6 Universidad Central de Venezuela, Caracas, Venezuela

## Abstract

**Background:**

The PLATINO project was launched in 2002 in order to study the prevalence of chronic obstructive pulmonary disease (COPD) in Latin America. Because smoking is the main risk factor for COPD, detailed data on it were obtained. The aim of this paper was to evaluate the prevalence of smoking and incidence of initiation among middle-aged and older adults (40 years or older). Special emphasis was given to the association between smoking and schooling.

**Methods:**

PLATINO is a multicenter study comprising five cross-sectional population-based surveys of approximately 1,000 individuals per site in Sao Paulo (Brazil), Santiago (Chile), Mexico City (Mexico), Montevideo (Uruguay) and Caracas (Venezuela). The outcome variable was smoking status (never, former or current). Current smokers were those who reported to smoke within the previous 30 days. Former smokers were those who reported to quit smoking more than 30 days before the survey. Using information on year of birth and age of smoking onset and quitting, a retrospective cohort analysis was carried out. Smoking prevalence at each period was defined as the number of subjects who started to smoke during the period plus those who were already smokers at the beginning of the period, divided by the total number of subjects. Incidence of smoking initiation was calculated as the number of subjects who started to smoke during the period divided by the number of non-smokers at its beginning. The independent variables included were sex, age and schooling.

**Results:**

Non-response rates ranged from 11.1% to 26.8%. The prevalence of smoking ranged from 23.9% (95%CI 21.3; 26.6) in Sao Paulo to 38.5% (95%CI 35.7; 41.2) in Santiago. Males and middle-aged adults were more likely to smoke in all sites. After adjustment for age, schooling was not associated with smoking. Using retrospective cohort analysis, it was possible to detect that the highest prevalence of smoking is found between 20–29 years, while the highest incidence is found between 10–19 years. Age of smoking onset tended to decline over time among females.

**Conclusion:**

The prevalence of smoking varied considerably across sites, but was lower among countries with national anti-smoking campaigns.

## Introduction

The Global Burden of Disease study forecasted that tobacco-attributable mortality would increase from 4.8 million deaths in 2000 to 8.4 million in 2020[1,2]. Declining trends in the prevalence of smoking among adult males are being offset by ascending trends for females and adolescents [3-6]. Large increases in smoking in developing countries were observed over the last part of the twentieth century[7]; China, for example, is experiencing a dramatic increase in smoking rates[3]. Trends in per capita cigarette consumption for adults between 1970 and 1992 varied depending of the level of development of the countries. In high-income countries, a reduction of 0.5% was observed, while an increase of 2.5% was found in low- and middle-income countries, leading to an overall worldwide increase of 0.8%[8].

Lopez et al[9] proposed a descriptive model of the cigarette epidemic in developed countries, including four stages, taking into account prevalence of smoking among adults, amount of cigarettes smoked per adult in a given period, and mortality related to smoking. By using this model, it is possible to identify the approximate stage of the smoking epidemic in a given country.

However, information on smoking prevalence in low and middle-income countries has been seriously limited by the lack of population-based studies using standardized methodology [10]. In 2002, the PLATINO study was launched with the main objective of measuring the prevalence of chronic obstructive pulmonary disease (COPD) and its risk factors in five Latin American metropolitan areas[11]. Detailed data on smoking were collected, giving us the opportunity to have standardized data on smoking across five Latin American areas. This paper presents prevalence of smoking and incidence of smoking initiation in Latin American middle-aged and older adults, using both cross-sectional and retrospective cohort analyses. Emphasis is given to the association between smoking and schooling.

## Methods

The PLATINO study was a multicenter survey carried out in five Latin American metropolitan areas: Sao Paulo (Brazil; survey took place in 2003), Mexico City (Mexico; survey took place in 2003), Montevideo (Uruguay; survey took place in 2004), Santiago (Chile; survey took place in 2004) and Caracas (Venezuela; survey took place in 2004). A cross-sectional design was used for obtaining representative samples of adults aged 40 years or older living in each city. In each of the sites, 68 census tracts (areas comprising approximately 300 households, which are delimited by national institutes of geography and statistics) were selected with probability proportionate to size, after being ranked according to socioeconomic indicators (schooling of family heads or household income). An average of 15 households was randomly selected in each tract. Face-to-face interviews were administered by trained interviewers to all residents aged 40 years or more living in the sampled households.

A standardized and pre-tested questionnaire was used (The Lung Health Study Questionnaire). We initially asked subjects whether or not they have smoked within the previous 30 days. Those who answered positively were classified as current smokers, regardless the amount reported. Information on number of cigarettes smoked per day was also collected among current smokers. Subjects who reported not to smoke in the 30 days prior to the interview were classified either as never smokers or former smokers. For current and former smokers, we asked the age of smoking onset. Former smokers also reported the age of quitting. Current use of pipe and cigars was also investigated. The independent variables included gender, age in years, and schooling (highest degree achieved). For subjects who refused to answer the whole questionnaire, we attempted to collect information on gender, age and current smoking statues for a better understanding of the characteristics of non-respondents.

All analyses were stratified by gender. Current smoking status was calculated according to age and schooling. The effect of years of schooling on smoking was adjusted for age (in three categories) using logistic regression. The odds ratio means the increment (or decrease) in the prevalence of smoking for each increased year of schooling. Using information on year of birth and age of smoking onset and quitting, a retrospective cohort analysis was carried out. Smoking prevalence at each period was defined as the number of subjects who started to smoke during the period plus those who were already smokers at the beginning of the period, divided by the total number of subjects. Incidence of smoking initiation was calculated as the number of subjects who started to smoke during the period divided by the number of non-smokers at its beginning. All analyses took the clustering of the sample into account, and were carried out using Stata 10.0.

Fieldwork lasted for approximately four months in each site, and the supervisors repeated 10% of the interviews within two weeks after the initial contact for quality control purposed. Individuals provided written informed consent prior to the interview and the protocol was approved by ethical boards in each country. Details of the methodology of the multicenter study are described elsewhere[12].

## Results

The numbers of eligible individuals were 1,150 in Sao Paulo, 1,476 in Santiago, 1,452 in Mexico City, 1,106 in Montevideo and 1,527 in Caracas. Non-response rates were 13.0%, 18.2%, 26.8%, 14.7% and 11.1%, respectively. For about half of all non-responders, it was possible to obtain information on age, gender and smoking status. Non-response was higher in men than women, tended to increase with age, and did not vary according to current smoking status[11]. More females than males were included in the sample due to a combination of increased life expectancy and lower non-response rates among females.

Table 1 describes the samples in terms of age and schooling stratified by gender. The percentage of females ranged from 55.8% in Sao Paulo to 65.1% in Caracas. The proportion of subjects aged 60 years or more was highest in Montevideo in both genders. Among men, low schooling was least common in Santiago and most frequent in Sao Paulo. For women, Montevideo and São Paulo had the highest and lowest schooling levels. All these differences across countries were highly statistically significant, but the composition of all samples was very similar to national census data .

**Table 1:** Description of the samples in the PLATINO study according to gender, age and schooling

	São Paulo		Santiago		Mexico City		Montevideo		Caracas	
	
Variable	Men	Women	Men	Women	Men	Women	Men	Women	Men	Women
	N(%)	N(%)	N(%)	N(%)	N(%)	N(%)	N(%)	N(%)	N(%)	N(%)
Age (years)										
40–49	174(39.5)	216(38.7)	161(34.6)	246(33.1)	169(39.2)	251(39.7)	105(27.6)	143(25.4)	181(40.1)	342(40.6)
50–59	149(33.8)	171(30.7)	152(32.7)	228(30.7)	116(26.9)	184(29.1)	103(27.1)	144(25.6)	132(29.3)	246(29.2)
≥ 60	118(26.8)	171(30.7)	152(32.7)	269(36.2)	146(33.9)	197(31.2)	172(45.3)	276(49.0)	138(30.6)	255(30.3)
Schooling (years)										
0–2	105(23.8)	128(23.1)	19(4.1)	68(9.2)	72(16.7)	132(20.9)	26(6.8)	36(6.4)	37(7.8)	110(12.5)
3–4	121(27.4)	187(33.7)	37(8.0)	82(11.0)	50(11.6)	74(11.7)	55(14.5)	91(16.3)	47(9.9)	114(12.9)
5–8	105(23.8)	121(21.8)	141(30.3)	220(29.6)	142(33.0)	207(32.8)	149(39.2)	218(38.6)	216(45.6)	365(41.3)
≥ 9	111(25.1)	119(21.4)	268(57.6)	373(50.2)	167(38.8)	218(34.6)	150(39.5)	216(38.6)	174(36.7)	294(33.3)
Overall (N)	442	558	465	743	431	632	380	563	474	883

The prevalence (95%CI) of smoking were 23.9% (21.3; 26.6) in Sao Paulo, 38.5% (35.7; 41.2) in Santiago, 25.4% (22.8; 28.0) in Mexico City, 28.0% (25.2; 30.9) in Montevideo and 28.5% (26.1; 30.9) in Caracas. The corresponding figures for males and females were, respectively, 30.2% and 19.0% in Sao Paulo (P < 0.001), 42.6% and 35.9% in Santiago (P = 0.02), 38.3% and 16.6% in Mexico City (P < 0.001), 33.3% and 24.5% in Montevideo (P = 0.003), 33.8% and 25.7% in Caracas (P = 0.002). Out of the 1,626 individuals classified as current smokers, only 2 (0.12%) reported not to be daily smokers.

The proportions of current smokers were virtually unchanged when the information for approximately one half of non-responders, who agreed to report their smoking status, were also considered. The prevalence of current use of cigar and pipe was 0.84% (47 of 5,566 subjects). Thirty two of these were from the top two schooling groups. No difference in the use of cigar or pipe was observed according to age. The lowest prevalence (0.3%) was observed in Chile, whereas the highest one (1.7%) was seen in Venezuela.

Table 2 presents the association between schooling, age and current smoking status among males. The prevalence of smoking in males decreased with age in all sites. Schooling was not associated with smoking in Sao Paulo, Santiago, Montevideo and Caracas, but in Mexico City there was a direct association. Table 3 shows that age was also inversely associated with current smoking among females, in all sites. Schooling showed no effect on smoking prevalence in Sao Paulo and Caracas, but in the other sites there was a direct association.

**Table 2:** Current smoking status in males from the five sites of the PLATINO study according to age and schooling

	Age (years)				Schooling (years)					
		
Smoking status	40–49	50–59	60 +	P	0–2	3–4	5–8	9 +	P	Total %
Sao Paulo (N = 442)				0.01					0.19	
Never smoker	32.2	21.5	23.9		23.1	21.5	35.2	26.1		26.3
Former smoker	33.3	49.7	50.4		45.2	51.2	35.2	41.4		43.5
Current smoker	34.5	28.9	25.6		31.7	27.3	29.5	32.4		30.2
										
Santiago (N = 465)				<0.001					0.97	
Never smoker	20.5	21.1	29.0		31.6	27.0	22.7	22.8		23.4
Former smoker	24.2	33.6	44.7		31.6	29.7	35.5	34.0		34.0
Current smoker	55.3	45.4	26.3		36.8	43.2	41.8	43.3		42.6
										
Mexico City (N = 431)				<0.001					0.03	
Never smoker	31.4	29.3	32.2		29.2	28.0	30.3	33.5		31.1
Former smoker	18.3	21.0	44.5		47.2	34.0	27.5	25.2		30.6
Current smoker	50.3	39.7	23.3		23.6	38.0	42.3	41.3		38.3
										
Montevideo (N = 380)				<0.001					0.07	
Never smoker	26.9	21.4	22.7		7.7	14.6	24.3	28.7		23.5
Former smoker	32.7	31.1	57.0		61.5	54.6	39.2	40.0		43.3
Current smoker	40.4	47.6	20.3		30.8	30.9	36.5	31.3		33.3
										
Caracas (N = 474)				<0.001					0.44	
Never smoker	35.9	26.5	17.4		21.6	19.2	26.9	30.5		27.0
Former smoker	22.1	38.6	60.9		40.5	53.2	38.0	36.8		39.2
Current smoker	42.0	34.9	21.7		37.8	27.7	35.2	32.8		33.8

^a ^Wald test

**Table 3:** Current smoking status in females from the five sites of the PLATINO study according to age and schooling

	Age (years)				Schooling (years)					
		
Smoking status	40–49	50–59	60 +	P	0–2	3–4	5–8	9 +	P	Total %
Sao Paulo (N = 558)				<0.001					0.31	
Never smoker	43.1	54.4	74.3		52.3	58.3	59.5	52.9		56.1
Former smoker	27.8	28.1	18.1		30.5	25.7	19.8	22.7		24.9
Current smoker	29.2	17.5	7.6		17.2	16.0	20.7	24.4		19.0
										
Santiago (N = 743)				<0.001					<0.001	
Never smoker	24.0	32.9	59.1		58.8	53.4	40.0	32.4		39.4
Former smoker	20.3	29.0	24.9		25.0	22.0	23.6	25.7		24.6
Current smoker	55.7	38.2	16.0		16.2	24.4	36.4	41.8		35.9
										
Mexico City (N = 632)				<0.001					<0.001	
Never smoker	66.5	72.3	79.7		79.6	90.5	73.0	61.5		72.3
Former smoker	9.6	10.3	13.7		9.1	4.1	12.1	13.8		11.1
Current smoker	23.9	17.4	6.6		11.4	5.4	15.0	24.9		16.6
										
Montevideo (N = 563)				<0.001					0.003	
Never smoker	35.0	48.0	71.4		72.2	67.0	59.3	45.4		56.1
Former smoker	24.5	24.3	14.1		11.1	12.1	19.4	23.6		19.4
Current smoker	40.6	27.8	14.5		16.7	20.9	21.3	31.0		24.5
										
Caracas (N = 883)				<0.001					0.75	
Never smoker	49.4	47.6	52.9		51.8	45.6	50.0	52.4		50.4
Former smoker	15.5	28.9	29.0		25.0	29.8	23.6	21.8		23.9
Current smoker	35.1	23.6	18.0		23.6	24.6	26.6	25.9		25.7

^a ^Wald test

The effect of age on the prevalence of smoking was unaltered after adjustment for schooling using logistic regression (data not shown). In Table 4, the crude and adjusted associations between smoking and schooling (highest degree achieved) are presented. After adjustment for age, schooling was directly associated with smoking among Montevideo and Santiago males, and inversely among Mexico City females. No other significant associations were observed. Tests for interaction between sex and schooling resulted in the following P levels: 0.03 in Mexico, 0.13 in Santiago, 0.14 in Montevideo, 0.77 in Brazil and 0.82 in Caracas. Thus, there is some evidence that in three of the sites, the association between smoking and schooling is different between the sexes.

**Table 4:** Adjusted^a ^odds ratio for current smoking according to years of schooling (numeric variable) in the five sites of the PLATINO study

**SITE**	**Men**	**Women**
	**Schooling (years)**	**Schooling (years)**

SAO PAULO		
Adjusted odds ratio (95%CI)	0.99 (0.95;1.03)	1.00 (0.96;1.05)
P (adjusted analysis)	0.56	0.94

SANTIAGO		
Adjusted odds ratio (95%CI)	0.95 (0.90;0.99)	1.01 (0.98;1.05)
P (adjusted analysis)	0.02	0.53

MEXICO CITY		
Adjusted odds ratio (95%CI)	0.99 (0.95;1.03)	1.05 (1.00;1.10)
P (adjusted analysis)	0.62	0.03

MONTEVIDEO		
Adjusted odds ratio (95%CI)	0.94 (0.89;0.99)	1.01 (0.96;1.06)
P (adjusted analysis)	0.03	0.74

CARACAS		
Adjusted odds ratio (95%CI)	0.96 (0.92;1.01)	0.97 (0.93;1.01)
P (adjusted analysis)	0.16	0.10

^a ^Adjusted for age (in three categories) using logistic regression taking the clustering of the sample into account.

Table 5 shows the prevalence of smoking and incidence of smoking initiation in each site according to age groups. Both for males and females, the highest prevalence was observed at 20–29 years, with few exceptions. The highest incidence of smoking initiation was observed between 10–19 years of age in men and women from all sites. Prevalence and incidence were identical in the first age range, and very similar in the second age group. Thereafter, prevalence values were consistently higher than incidence ones. The median age of smoking onset was higher among women than men in all sites. For men, the lowest median was observed in Montevideo for women in São Paulo. For both genders, the highest median age of smoking onset was observed in Mexico City.

**Table 5:** Smoking prevalence and incidence by age group and gender in the PLATINO study: retrospective cohort analyses.

Age (years)	Measure	Sao Paulo		Santiago		Mexico City		Montevideo		Caracas	
		
		Men	Women	Men	Women	Men	Women	Men	Women	Men	Women
0–9	Prevalence^a^	6.3	1.8	2.8	1.5	3.0	0.3	2.6	0.4	0.9	0.6
	Incidence^b^	6.3	1.8	2.8	1.5	3.0	0.3	2.6	0.4	0.9	0.6
											
10–19	Prevalence	57.6	31.0	58.7	35.9	47.3	12.3	65.3	27.9	61.2	32.0
	Incidence	54.9	29.7	57.6	35.0	45.8	12.1	64.3	27.6	60.9	31.6
											
20–29	Prevalence	68.5	38.5	69.9	51.8	59.9	20.9	72.9	35.7	73.4	46.0
	Incidence	29.4	12.9	30.0	26.3	27.6	10.2	25.4	11.5	33.3	21.1
											
30–39	Prevalence	61.9	33.0	63.2	49.7	60.1	23.9	67.1	35.7	65.2	44.8
	Incidence	4.5	2.3	6.6	7.9	8.0	5.3	3.8	5.0	3.7	6.3
											
40–49	Prevalence	50.1	27.4	57.4	47.2	53.8	23.3	59.2	33.9	56.1	39.5
	Incidence	1.5	1.3	4.9	5.6	5.8	2.2	1.0	5.7	2.7	2.9
											
50–59	Prevalence	41.6	18.1	46.7	36.0	46.9	18.1	50.2	26.2	47.4	31.7
	Incidence	1.2	2.0	3.4	3.3	2.4	1.8	1.2	0.8	0.0	2.2
											
≥ 60	Prevalence	39.8	41.5	32.2	20.8	40.4	12.7	37.2	19.2	40.6	26.7
	Incidence	0.0	17.1	3.6	0.0	0.0	1.3	0.0	4.5	0.0	0.0
											
Median age at starting smoking (years)		16.2	18.3	17.4	20.0	18.2	23.3	16.0	21.1	17.0	19.8

^a ^Number of subjects who started to smoke during the period plus those who were already smokers at the beginning of the period divided by the total number of subjects in the period.

^b ^Number of subjects who started to smoke during the period divided by the number of non-smokers at the beginning of the period.

**Figure 1:**
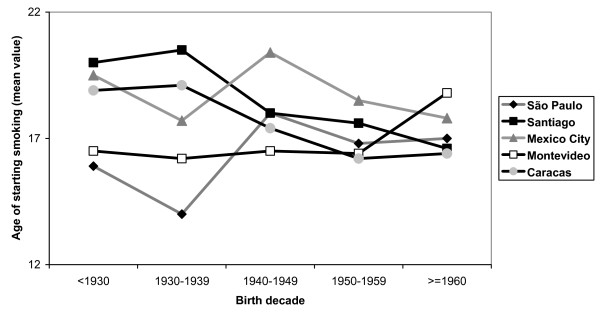


**Figure 2:**
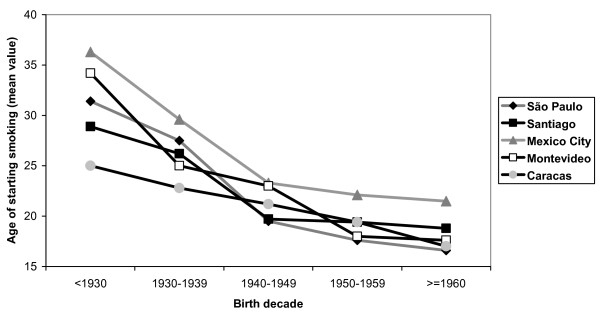


Figure 1 presents the mean age of smoking onset in men according to birth cohorts. Trends were not consistent across sites. In Caracas and Santiago, declining trends were observed (P for linear trend < 0.001). For São Paulo and Mexico City, the lowest mean age was observed for those born between 1930–39, and the highest for the 1940–49 cohort, but the overall trend was not clear. In Montevideo, no variations in the age of smoking onset were observed for those born before 1960, after which there was a marked increase. Unlike men, women (Figure 2) showed consistent patterns across sites, with those born before 1930 presenting the highest mean ages of smoking onset, with declining trends thereafter.

## Discussion

Tobacco use continues to be a leading preventable cause of death and disability among adults worldwide, accounting for 4.1% of all DALYs lost[13], and is the main cause of cancer deaths both in high-income countries and in low- and middle-income settings[14]. In the year 2000, low- and middle-income countries accounted for 71% of global tobacco consumption[15]. The Research for International Tobacco Control Network, formerly the International Tobacco Initiative, highlighted the lack of standardized and comparable data on smoking in these countries[10]. Local data on smoking patterns are essential for the development of tobacco control policies and programs, as well as the increase on funding for tobacco control research. There are very few multicenter population-based data on smoking in Latin America, and some of the data available are restricted to specific age groups [16].

The present study helps fill the gap on standardized data on smoking in Latin America, using representative samples of the largest metropolitan areas in each of the five countries. Response rates were relatively high, and there was no evidence that non-responders differed from those who answered the full questionnaire in terms of current smoking status[11]. The possibility that our findings are due to confounding was minimized by the use of multivariable techniques.

The prevalence of smoking varied considerably among the five cities included in the PLATINO study. Understanding these differences can be important for planning effective interventions against smoking. It is not surprising that the two countries (Chile and Uruguay) which did not have national actions plans against smoking at the time of the surveys presented the highest prevalence of smoking .

Effective interventions against smoking are needed in Santiago, where smoking prevalence was highest for both men and women. Men, but not women, in Mexico City also present very high prevalence of smoking. Because of important differences between the five sites, pooled analyses were not attempted. In addition, examination of the prevalence of smoking in a given period alone was shown to be an insufficient indicator of the stages of the tobacco epidemic, because the peak of the harmful effects of smoking on health are only detectable 20–40 years after the peak of the smoking prevalence[9]. Using the stages proposed by Lopez et al[9], the five sites included in the PLATINO study appear to be at different stages of the tobacco epidemic, probably due to different legislations and effectiveness of anti-smoking strategies. Broadly, Latin America was considered as being in stage II of the tobacco epidemic[9].

We did not find a clear association between schooling and smoking prevalence after adjustment for age (Table 4), but there is evidence of an inverse association among males in the two sites with highest smoking prevalence (Santiago and Montevideo). There is also evidence of a positive association with schooling among women in the site with the lowest prevalence, Mexico City. There was a significant interaction between schooling and sex in Mexico (P = 0.03), and the P values for this interaction were below 0.20 in Santiago and Montevideo. One may hypothesize whether in groups where the epidemic has reached its peak – e.g. males – those with high schooling have already quitted smoking; whereas where the epidemic is still growing – e.g. females – smoking is still more common among those with high schooling. This hypothesis is consistent with the literature[3,5].

It was previously shown that current prevalence of smoking alone is an insufficient indicator of cumulative risk[1,17]. The retrospective cohort analyses showed that the age groups in which smoking prevalence was highest were not the same that had the highest incidence of initiation. This finding has policy implications. Specific campaigns for preventing smoking onset should focus adolescents, who present the highest incidence values. On the other hand, specific campaigns for quitting smoking should address older subjects, who present the highest prevalence values.

Our findings showed a consistent reduction in the mean age of smoking onset in females over time (at least until the 70's), whereas there was no clear pattern for males. Other studies suggest that adolescent smoking is a growing problem and that risk factors for smoking are different between boys and girls[5,6,18]. All these differences across the sexes should be taken into account when planning anti-smoking interventions.

Some limitations of this study should be considered. First, given the focus of the PLATINO project on chronic obstructive pulmonary disease (COPD), individuals aged less than 40 years were not included. This was done because COPD rates are particularly concerning among middle-aged and older adults, and not among younger subjects. Also, our results cannot be extrapolated to Latin America as a whole, because only large urban metropolitan areas were studied and adolescents or young adults were not eligible. Third, there is a possibility of survival bias in the retrospective cohort analyses (Figures 1 and 2); older subjects who started to smoke at a young age may have died, and part of the declining trend in the age of starting smoking may be explained by this bias. However, because such trends are more marked for women, who are less likely to die prematurely, the observed trends are unlikely to be solely due to survival bias. Also, some degree of overestimation in the prevalence of former smoking is expected, because 30 days may be a short period for true abstinence. Another issue is that face-to-face interviews at home may not warrantee privacy to disclose smoking. Finally, it is important to remember that the trends in the mean age of smoking onset reflect the situation of the studied countries some decades ago, and care should be paid for extrapolating these findings to nowadays.

## Conclusion

The different tobacco legislation, policies and interventions existing in each of the countries studied may explain some of the differences presented in this paper. Brazil, Mexico and Venezuela, which present national actions plans aiming at reducing the burden of smoking presented the lowest values. Data presented in this publication allow formal comparisons of smoking prevalence, incidence of initiation and risk factors across Latin America countries. Policy makers in each setting may use the information presented here in order to plan effective interventions.

## Competing interests

The authors declare that they have no competing interests.

## Authors' contributions

AM, PH and CV led the analyses and writing processes. RP coordinated the study in Mexico. AM and ML coordinated the study in Uruguay. GV and JP coordinated the study in Chile. JJ coordinated the study in Brazil. MO and CT coordinated the study in Venezuela. All authors contributed to early drafts of this manuscript and approved its final version.

## Pre-publication history

The pre-publication history for this paper can be accessed here:


